# Synthesis and Characterization of Multifunctional Nanovesicles Composed of POPC Lipid Molecules for Nuclear Imaging

**DOI:** 10.3390/molecules26216591

**Published:** 2021-10-30

**Authors:** Debora Petroni, Claudia Riccardi, Domenico Cavasso, Irene Russo Krauss, Luigi Paduano, Daniela Montesarchio, Luca Menichetti

**Affiliations:** 1 Institute of Clinical Physiology, National Research Council, Via Moruzzi 1, I-56124 Pisa, Italy; luca.menichetti@ifc.cnr.it; 2 Department of Chemical Sciences, University of Naples Federico II, Via Cintia 21, I-80126 Naples, Italy; claudia.riccardi@unina.it (C.R.); domenico.cavasso@unina.it (D.C.); irene.russokrauss@unina.it (I.R.K.); luigi.paduano@unina.it (L.P.); daniela.montesarchio@unina.it (D.M.); 3 CSGI-Consorzio per lo Sviluppo dei Sistemi a Grande Interfase, I-50100 Florence, Italy

**Keywords:** liposome, PET, gallium-68, drug delivery, POPC, NOTA

## Abstract

The integration of nuclear imaging analysis with nanomedicine has tremendously grown and represents a valid and powerful tool for the development and clinical translation of drug delivery systems. Among the various types of nanostructures used as drug carriers, nanovesicles represent intriguing platforms due to their capability to entrap both lipophilic and hydrophilic agents, and their well-known biocompatibility and biodegradability. In this respect, here we present the development of a labelling procedure of POPC (1-palmitoyl-2-oleoyl-sn-glycero-3-phosphocholine)-based liposomes incorporating an ad hoc designed lipophilic NOTA (1,4,7-triazacyclononane-1,4,7-triacetic acid) analogue, derivatized with an oleic acid residue, able to bind the positron emitter gallium-68(III). Based on POPC features, the optimal conditions for liposome labelling were studied with the aim of optimizing the Ga(III) incorporation and obtaining a significant radiochemical yield. The data presented in this work demonstrate the feasibility of the labelling procedure on POPC liposomes co-formulated with the ad hoc designed NOTA analogue. We thus provided a critical insight into the practical aspects of the development of vesicles for theranostic approaches, which in principle can be extended to other nanosystems exploiting a variety of bioconjugation protocols.

## 1. Introduction

Among the various types of known drug carriers, the nanostructured ones (micelles, dendrimers, etc.)—by far the most investigated delivery systems [1]—find application also in the radiopharmaceutical field. Indeed, nuclear imaging techniques are frequently used to study new drug delivery systems because of their unique capacity to perform dynamic studies [2,3]. Liposomes are a notable exception in this context: their limited use as radiopharmaceuticals is mainly related to their intrinsic properties and peculiar physical and chemical stability. Liposomes are phospholipid-based nanovesicles, with dimensions ranging from dozens of nanometres to several microns, formed by single or multiple concentric lipid bilayers with an internal aqueous core, showing a morphology very similar to cellular membranes [4]. The different interplay between hydrophobic and hydrophilic layers in their microstructure makes labelling of liposomes a real challenge; in fact, the control of the labelling efficiency and stability of the radiolabelled product is a critical issue.

The aim of this work is the design and preparation of stable liposomal formulations suitable for positron emission tomography (PET) imaging.

PET imaging is a powerful method to visualize in real-time and quantify biochemical events in living tissues and intact organs, providing a fast and non-invasive evaluation of physiological and pathological processes [2,5,6]. Indeed, PET has become a well-established clinical imaging technique. It also plays an important role in drug development and evaluation, whose crucial steps are understanding the mechanism of action and establishing the optimal dosage regimens and treatment strategies [7,8].

To detect liposomes by PET, their structure must be modified to allow incorporation of a suitable positron emitter radionuclide, such as ^68^Ga, ^64^Cu, ^18^F, etc. A selected list of the most-used PET radionuclides, along with their most relevant features, is reported in Table S1 [9]. The choice of the radionuclide depends on the properties of the selected nanostructure and the intended specific application.

We identified ^68^Ga as the positron emitter radionuclide suitable for our purposes based on its advantages over the other positron-emitting radionuclides.

First of all, it can be produced from cost-effective generators and therefore its use is more easily manageable by nuclear medicine departments, differently from most radionuclides listed in Table S1, whose use is limited to facilities equipped with cyclotron [10]. Other important aspects are the value of its half-life (t_1/2_ = 68 min), compatible with clinical protocols, and the high quality of images obtained in a short scanning time and therefore with a lower radiation dose for patients and personnel.

In addition, the remarkable versatility in labelling chemistry and the reactivity in chelation reactions represent other significant characteristics underpinning the candidate’s selection. Moreover, the simplicity of some labelling reactions amenable to kit type preparation and the potential for theranostics have recently emerged [11,12,13,14,15,16,17,18]. Hence, the use of ^68^Ga is growing rapidly, both in clinical research and in routine diagnostic imaging.

Several approaches are currently available to label a liposome, either based on encapsulation of the radionuclide in the inner part of the vesicle (remote loading or passive encapsulation) or on surface labelling. The latter strategy can be performed by membrane labelling (the radionuclide is inserted within the lipid bilayer) or by using suitable bifunctional chelating agents, incorporated into the liposome surface or chemically linked to it as such or through a proper spacer [19]. The choice of the labelling method depends on the characteristics of both the nanostructures and the radionuclide.

To the best of our knowledge, only few ^68^Ga-labelled liposomes have been prepared [15,18]. To expand the potential and applicability of these systems, in this work, we describe a new synthesis and labelling approach starting from a POPC liposome (1-palmitoyl-2-oleoyl-sn-glycero-3-phosphocholine). We selected POPC-based liposomes as the carrier for ^68^Ga on the basis of their well-proven biocompatibility [20,21,22] and their effective use in a broad range of biotechnological applications [23]. Moreover, we have good expertise with these nanostructures, which we already exploited in the development of suitable metal-based drug delivery systems [24,25,26].

Several macrocyclic chelators, such as NOTA (1,4,7-triazacyclononane-1,4,7-triacetic acid) and DOTA (1,4,7,10-tetraazacyclododecane-1,4,7,10-tetraacetic acid), are available for gallium complexation [27,28]. The thermodynamic constant of the Ga^3+^ complex with the DOTA chelator is much lower (logK = 21.33) [29] than that of its NOTA analogue (logK = 30.98) [30]. Therefore, NOTA was selected in this study as the host macrocycle.

Thus, we incorporated an ad hoc designed lipophilic NOTA analogue derivatized with an oleic acid residue—that we named NOTA-OL—in POPC liposomes in order to efficiently coordinate the positron emitter nuclide gallium-68(III). In detail, NOTA-OL consists of a polar NOTA moiety, a flexible linker based on 1,2-diaminoethane, and an oleic acid residue. The lipophilic linear 18-carbon atom chain ensures the insertion of NOTA-OL in the lipid bilayer of POPC liposomes by exploiting hydrophobic interactions. Stable amide bonds were chosen to connect the three modular parts of the NOTA derivative, because they can be easily realized and are chemically stable under a wide range of conditions, particularly in the acidic solutions used for the protective group removal during synthesis.

The lipophilic derivative NOTA-OL was then co-formulated with POPC to obtain stable liposomes; several protocols were tested for their labelling with ^68^Ga and the experimental conditions were optimized.

## 2. Materials and Methods

All the reagents and solvents were of the highest commercially available quality and were used as received. NODA-Ga(*t*Bu)_3_ was purchased from CheMatech (Dijon, France). 1,2-Diaminoethane, oleic acid, and Ga(NO_3_)_3_ were provided by Sigma-Aldrich (Milan, Italy).

Thin-layer chromatography (TLC) analyses were performed on pre-coated silica gel 60 plates with fluorescent UV_254_ indicator supplied by Macherey-Nagel (Düren, Germany). TLC sheets were visualized using a UV-light lamp and then treated with an oxidant acidic solution (acetic acid, water, and sulfuric acid, 10:4:5, *v/v*). For the purification of the here synthesized compounds by column chromatography, unmodified silica gel 60 (particle size: 0.063–0.200 mm; pore size: 60 Å) was used (Macherey-Nagel).

NMR spectra were acquired using a Bruker WM-400 spectrometer (Bruker, Billerica, MA, USA). All the chemical shifts (*δ*) are expressed in ppm with respect to the residual solvent signal both for ^1^H NMR (CDCl_3_ = 7.26 ppm) and ^13^C NMR (CDCl_3_ = 76.9 ppm). All the coupling constants (*J*) are quoted in Hertz (Hz). Peak assignments were carried out based on standard ^1^H-^1^H COSY and HSQC experiments (Supplementary Material).

MALDI-TOF mass spectrometric investigations were carried out on a TOF/TOF™ 5800 system (AB Sciex, Framingham, MA, USA) according to the reported procedures [31].

HPLC chromatographic purification and analysis of NOTA-OL were carried out on an Agilent HPLC system (Santa Clara, CA, USA), supplied with a UV/vis detector, by using a Kinetex C18 column (250 × 10 mm, 5 µm, Phenomenex, Torrance, CA, USA). The HPLC column elution—monitored at λ = 218 nm—was performed using gradients of CH_3_OH 0.1% TFA (solution B) in H_2_O 0.1% TFA (solution A) and a flow rate of 0.8 mL min^−1^.

The surface tension, γ, of the aqueous mixture containing NOTA-OL was measured by the De Nouy ring method using a KSV Sigma 70 digital tensiometer, (KVS, Helsinki, Finland) [32]. Weighed amounts of a NOTA-OL solution at a concentration well above its critical micelle concentration (cmc) were added to a weighed amount of aqueous solution in the tensiometer circular vessel at 25 °C.

The UV-vis experiments were carried out on a V-530 UV-vis spectrophotometer (JASCO, Italy), equipped with a JASCO ETC-505T Peltier Thermostat, by using a quartz cuvette with a path length of 1 cm (Hellma). The UV spectra, subtracted off the appropriate baseline, were recorded at r.t. with the following parameters: medium response, scanning speed of 100 nm/min, 2.0 nm bandwidth, and 200–800 nm range. All the spectra were averaged over 3 scans and each experiment was performed in triplicate.

Dynamic light scattering (DLS) measurements were performed at the scattering angle θ = 90°, by using a home-made instrument composed of a Photocor compact goniometer, (Photocor Ltd., Moscow, Russia) a SMD 6000 Laser Quantum 50 mW light source operating at 532.5 nm, a photomultiplier (PMT120-OP/B), and a correlator (Flex02-01D) from Correlator.com. All the measurements were performed at 25 °C with the temperature controlled through a thermostatic bath [33].

Small-angle neutron scattering (SANS) measurements were performed on a D11 diffractometer at the steady-state reactor source ILL (Grenoble, France) [34]. Measurements were performed at a constant neutron wavelength (λ) of 6 Å and sample-detector distances of 1.2 and 8 m to cover a q range between 0.008 and 0.5 Å^−1^.

The quality control of radiolabelled compounds was performed by using a radio-HPLC and radio-TLC analyses. The Waters (Milford, MA, USA) HPLC system (Delta 600 pump, injection loop 20 μL) was equipped with a photodiode array detector (996 PAD) and a NaI (Tl) scintillation detector (Gabi, Elysia-Raytest GmbH, Straubenhardt, Germany). Analyses were performed using a X-Terra MS C18 column (Waters, 100 × 3.0 mm, 5 μm) with a CH_3_OH 0. 1% TFA/H_2_O 0.1% TFA 80/20 (*v/v*) solution used as the mobile phase at a 1.0 mL min^−1^ flow rate.

The radio-TLC analyses were performed using a MiniGITA TLC scanner (Elysia-Raytest), ITLC-SG plates (Whatman, Maideston, United Kingdom), and citrate buffer (0.1 M, pH = 5) as the mobile phase.

### 2.1. Synthesis of NOTA-OL

#### 2.1.1. Synthesis of *N*-1-[(4-methoxyphenyl)diphenylmethyl]ethane-1,2-diamine (**2**)

**Figure 1:**
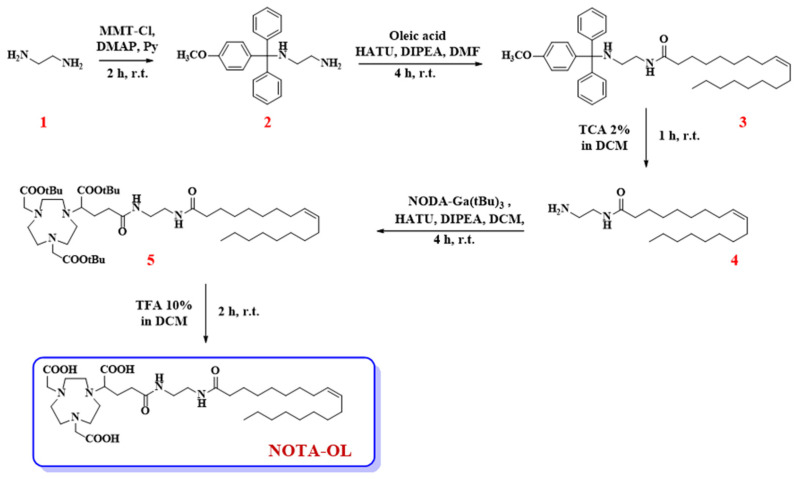
Synthetic procedure for the preparation of the here proposed NOTA-OL derivative.

First, 1,2-diaminoethane (**1**, 1.07 mL, 16 mmol) was dissolved in anhydrous pyridine (3.5 mL) and then DMAP (19.6 mg, 0.16 mmol) and MMT-Cl (248 mg, 0.80 mmol) were sequentially added to the reaction mixture. After 2 h under magnetic stirring at r.t., TLC monitoring denoted the presence of a new product in the reaction mixture. Thus, the reaction was blocked by adding few drops of CH_3_OH, the resulting mixture was concentrated under vacuum, and then DCM/H_2_O extraction was performed. The organic phases were recovered, dried over anhydrous Na_2_SO_4_, filtered, and lastly concentrated under vacuum. The dried residue was then purified by silica gel column chromatography using a mixture of DCM/MeOH (95:5, *v/v*) as eluent. This solution was also supplied with 2% *v/v* of triethylamine (TEA), used to avoid undesired detachment of the acid-labile MMT-protecting group. Target compound **2** was thus recovered as a pale-yellow oil in 75% overall yield (200 mg, 0.60 mmol) (Figure 1).

#### 2.1.2. Synthesis of *N*-2-[(4-methoxyphenyl)diphenylmethylamino]ethyl oleamide (**3**)

Oleic acid (116 μL, 0.36 mmol), HATU (260 mg, 0.68 mmol), DIPEA (159 mg, 0.91 mmol), and the previously synthesized compound **2** (151 mg, 0.45 mmol) were dissolved in anhydrous DMF (3 mL) and the resulting solution taken at r.t. under argon atmosphere. After 4 h, TLC monitoring of the reaction mixture indicated the complete disappearance of the starting oleic acid and the appearance of a more apolar product. Thus, CH_3_OH was added to quench the reaction and the solvent was removed under vacuum. Then, the crude product was purified by silica gel column chromatography, using *n*-hexane/AcOEt (8:2, *v/v*) as eluent containing 2% of TEA, providing the desired compound **3** as a pale-yellow oil in a 47% isolated yield (99.2 mg, 0.17 mmol).

#### 2.1.3. Synthesis of *N*-(2-aminoethyl)oleamide (**4**)

Pure compound **3** (32.3 mg, 0.05 mmol) was treated with 1 mL of a 2% TCA solution in DCM. This acid treatment produced an intense yellow-orange coloration in the reaction mixture, due to the formation of the MMT cation. After 1 h, compound **3** was completely consumed and a new more polar compound was detected in the reaction mixture, as indicated by the analysis on the TLC plates. To quench the reaction, CH_3_OH and then TEA were added to the mixture until a neutral pH was reached. After extraction with DCM/H_2_O, the crude residue recovered in the organic phases was purified by chromatography on a silica gel column eluting with DCM/CH_3_OH (98:2, *v/v*) containing 2% of TEA. The desired compound **4** was thus obtained in a pure form as a pale-yellow oil in a 98% yield (16.2 mg, 0.05 mmol).

#### 2.1.4. Synthesis of (*Z*)-di-tert-butyl 2,2′-[7-(1-tert-butoxy-5-2-oleamidoethylamino-1,5-dioxopentan-2-yl)-1,4,7-triazonane-1,4-diyl]diacetate (**5**)

NODA-Ga(*t*Bu)_3_ (37.1 mg, 0.07 mmol) was dissolved in anhydrous DCM (1 mL) and then treated with HATU (39.0 mg, 0.10 mmol) and DIPEA (23.8 mg, 0.14 mmol) as condensing agents, and finally with the previously obtained amine **4** (26.0 mg, 0.08 mmol). The reaction mixture was thus left under stirring and argon flow at r.t. After 4 h, TLC analysis indicated the complete consumption of both starting compounds as well as the appearance of a new product. Thus, the solvent was removed under vacuum and pure compound **5** was obtained as a yellow oil and in a 57% overall yield (33.4 mg, 0.04 mmol) after purification by chromatographic column eluting with CHCl_3_/CH_3_OH (95:5, *v/v*).

#### 2.1.5. Synthesis of NOTA-OL

Pure compound **5** (34.0 mg, 0.04 mmol) was dissolved in anhydrous HCOOH (1 mL) and left under stirring at r.t. until TLC analysis indicated the complete disappearance of the starting compound. After 2 h, the solvent was removed under vacuum and the crude purified by RP-HPLC on a C18 column with UV detection at 218 nm. For the elution, a gradient from 50 to 100% in 15 min of solution B in solution A (solution A: H_2_O, containing 0.1% TFA; solution B: CH_3_OH, containing 0.1% TFA) was used. By collecting the peak at t_R_ = 11.6 min, the pure desired NOTA-OL was recovered as an oil with an 80% yield (21.8 mg, 0.03 mmol).

### 2.2. Preparation of (Ga)NOTA-OL Complex

NOTA-OL (2.3 mg, 3.4 μmol) was dissolved in 2.3 mL of Milli-Q water and then 113 μL of a 30 mM Ga(NO_3_)_3_ solution (0.9 mg, 3.4 μmol) in Milli-Q water were added. Then, the pH of the mixture was adjusted to pH 2–4 by the addition of proper volumes of 0.1 M HCl solution. The reaction mixture was left under stirring at 50–60 °C and then at r.t. overnight, monitoring the formation of the desired complex by acquiring MALDI-TOF spectra. The (Ga)NOTA-OL complex was then purified from the unreacted NOTA-OL using a RP18-cartridge eluted with H_2_O/CH_3_OH solutions using increasing amounts of CH_3_OH. In detail, 7 mL of H_2_O/CH_3_OH (8:2, *v/v*), 7 mL of H_2_O/CH_3_OH (1:1, *v/v*), and 7 mL of H_2_O/CH_3_OH solutions (1:9, *v/v*) were progressively used as eluents.

All the collected fractions were analyzed by UV-vis and MALDI-TOF analyses. The target (Ga)NOTA-OL complex (1.0 mg, 1.34 µmol) was recovered with an overall yield of 40%.

### 2.3. NOTA-OL/POPC Formulations: Preparation

POPC liposomes loaded with NOTA-OL were prepared by the thin film protocol. Weighed amounts of both components, in order to obtain the prefixed 10:90 or 5:95 NOTA-OL/POPC molar ratio, were dissolved in chloroform and then mixed. The resulting solution was transferred into a round-bottom glass tube and the solvent was evaporated with anhydrous nitrogen to obtain a homogeneous thin film. To ensure the complete solvent removal, the samples were kept under vacuum for at least 24 h before rehydration. The samples were then hydrated with pure water, previously filtered through 0.22 µm filters, to obtain a total lipid concentration of 1 mM. Liposomes thus obtained were vortexed, briefly sonicated, and then extruded through polycarbonate membranes with 100 nm pores at least 15 times in order to obtain monodispersed unilamellar liposomes [35].

### 2.4. (Ga)NOTA-OL/POPC Preparation

Starting from preformed 1 mM NOTA-OL/POPC 5:95 liposomes, the amount of Ga(NO_3_)_3_ required to obtain the desired 1:1 mol ratio with NOTA-OL was added to the mixture. After acidification of the solution to reach pH in the range 3–4, the reaction mixture was left under stirring at 50–60 °C and then at r.t. overnight. The obtained liposomes were then analyzed as such by DLS.

### 2.5. ^68^Ga Production, [^68^Ga]NOTA-OL, and [^68^Ga]NOTA-OL/POPC Preparation

The radioisotope was produced as [^68^Ga]GaCl_3_ by a ^68^Ge/^68^Ga generator (Eckert &Ziegler, Berlin, Germany) using a 0.1 N HCl solution as eluent. Collected volumes (about 2.0 mL) were purified by solid phase extraction using a 30 mg/mL Strata X-C-33μ (Phenomenex) polymeric strong cationic exchange cartridge, eluting with a 0.02 N HCl/acetone (2:98, *v/v*) solution. Recovered fractions (600–700 μL) were concentrated under nitrogen flow.

To 10 μL of 1 mM of NOTA-OL solution, 90 μL of ^68^Ga^3+^ solution (23–27 MBq) were added and then the mixture was heated at 70 °C for 30 min. Radiochemical yield: 98% ± 2%.

Radio-HPLC: ^68^Ga^3+^ t_R_ ~ 0.7 min; [^68^Ga]NOTA-OL t_R_ ~ 4.8 min.

For liposome labelling, 75 µL of the ^68^Ga^3+^ solution (14 ÷ 19 MBq) were added to 200 μL of a 1.0 mM solution of NOTA-OL/POPC (containing 10% NOTA-OL in mol). The mixture (pH ~ 3.5) was heated at 60-70 °C for 40 min. Radiochemical yield: 56.6% ± 10.8%.

Radio-HPLC: ^68^Ga t_R_ ~ 0.7 min; [^68^Ga]NOTA-OL/POPC t_R_ ~ 4.5 min.

Radio-TLC: ^68^Ga^3+^ R_f_ ~ 0.9; [^68^Ga]NOTA-OL/POPC R_f_ ~ 0.1.

The reaction crude was purified on Strata X-C-33µ (Phenomenex) cationic exchange cartridges. The pure product was recovered by eluting the column with a solution of saline/0.02 N HCl (2%) and acetone (98%, *v/v*) 65/35 (three fractions of 500 μL).

## 3. Results and Discussion

### 3.1. Synthesis and Characterization of NOTA-OL

NOTA-OL was prepared through a simple and easily reproducible synthetic scheme (Figure 1) and then characterized by UV, NMR, and MALDI experiments.

The first reaction involved the mono-protection of compound **1** with the 4-monomethoxytrityl (MMT) protective group, producing the key intermediate **2** (Figure 1). The introduction of the protective group was performed to increase the yields of the successive coupling steps. In fact, in all our initial attempts to directly couple **1** with a carboxylic acid (either with oleic acid or with the tris-*t*Bu-protected NOTA derivative), a complex mixture was always obtained, mainly containing dimeric products. For this reason, MMT was introduced at one end of 1,2-diaminoethane. In this manner, a useful UV-visible derivative was obtained, carrying a unique reactive amino group available for the following coupling with oleic acid, which allowed simplification of the successive purification steps.

The tritylation reaction was hence performed by reacting 4-methoxytriphenylmethyl chloride (MMT-Cl) with an excess of 1,2-diaminoethane, necessary to favor the formation of the mono-alkylated product. Then, compound **2** was coupled with oleic acid in the presence of HATU and DIPEA as condensing agents, leading to compound **3**. The MMT-protecting group was then removed from compound **3** under acidic conditions, producing the desired primary amine **4**, which was lastly condensed with the commercially available NODA-Ga(*t*Bu)_3_, yielding the target compound **5**.

In the last step of our synthetic scheme, compound **5** was treated with formic acid to detach the *t*Bu-protecting groups. The complete *t*Bu removal from compound **5** was confirmed by TLC and NMR analysis. In detail, the ^1^H NMR spectra of the final compound showed the disappearance of the typical signal of *t*Bu protons. The desired compound was recovered after purification by RP-HPLC, and its purity and identity were ascertained by analytical RP-HPLC and MALDI-TOF MS analyses (Figures S1 and S2, respectively).

Following this synthetic scheme, target compound NOTA-OL was obtained in 5 steps and a 16% overall yield. All the synthetic intermediates and NOTA-OL were fully characterized using NMR spectroscopy and mass spectrometry techniques.

UV-vis analysis showed for NODA-Ga(*t*Bu)_3_ the presence of one main band centered at 218 nm, essentially due to the absorption features of the nitrogenated macrocycle (Figure S3, green line). In the case of NOTA-OL, this band was slightly blue-shifted, centered at 212 nm, and accompanied by another weak band, centered at about 280 nm (Figure S3, red line), characteristic of amide bonds. The self-aggregation behavior of NOTA-OL was investigated in aqueous solution to determine its cmc (Figure S4).

### 3.2. Synthesis of (Ga)NOTA-OL Complex

To verify its capability to chelate gallium ions, NOTA-OL was reacted with the Ga(NO_3_)_3_ salt, using conditions (low amount of reagents, small volumes, acidic pH, etc.) as close as possible to those generally exploited during a radiolabelling procedure with Gallium-68.

**Figure 2:**
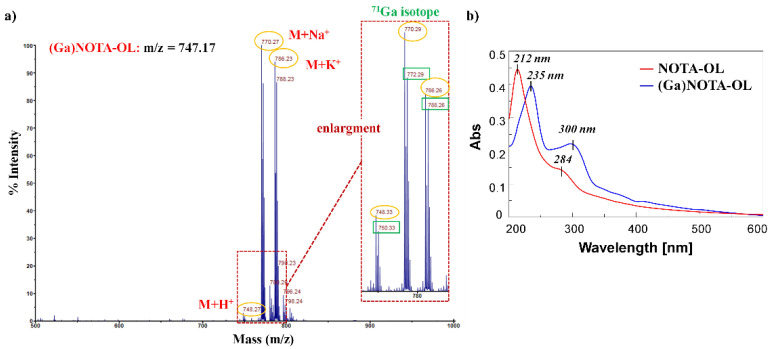
(**a**) MALDI-TOF spectrum (positive ions) of pure (Ga)NOTA-OL complex using 2,5-dihydroxybenzoic acid (DHB) as the matrix. The inset represents an enlargement of the 740–800 nm mass spectrum range; (**b**) Overlapped UV-vis absorption spectra of NOTA-OL (red line) and (Ga)NOTA-OL complex (blue line) at a 100 μM concentration, respectively in CH_3_CN and 1:1 CH_3_OH/H_2_O solutions.

The formation of the desired (Ga)NOTA-OL complex was monitored by MALDI-TOF MS analysis (Figure S5). Peaks with the expected m/z ratios for the (Ga)NOTA-OL complex were observed, but also peaks corresponding to unreacted NOTA-OL were present in the crude reaction mixture. In order to obtain the desired (Ga)NOTA-OL complex in a pure form, the mixture was purified using RP18-cartridges and the resulting (Ga)NOTA-OL complex was characterized by MALDI-TOF and UV-vis analysis (Figure 2).

MALDI-TOF MS analysis confirmed the exclusive presence of the desired (Ga)NOTA-OL complex without peaks attributable to the starting NOTA-OL compound (Figure 2a). Interestingly, in the UV-vis spectrum of (Ga)NOTA-OL, both the absorption bands of NOTA-OL (the main one at 212 nm and the weaker one at ca. 280 nm (Figure 2b, red line)) were red-shifted, being respectively centered at ca. 235 and 300 nm (Figure 2b, blue line). These UV differences, also taking into account properly subtracted solvent effects, were diagnostic of metal complexation.

### 3.3. Synthesis of [^68^Ga]NOTA-OL Complex

The same procedure used for the synthesis of (Ga)NOTA-OL was then applied for the complexation of NOTA-OL with the radioactive ^68^Ga in order to verify the efficacy of the labelling method and set up the optimal quality control conditions. In this case, to increase the overall yield and reduce the reaction time, the complexation was performed at a higher temperature (70 °C) for 30–40 min. Radio-HPLC chromatograms showed that the reaction between NOTA-OL and ^68^Ga took place with an excellent radiochemical yield, higher than 98%.

Once the feasibility of the complexation reaction of NOTA-OL was verified both with non-radioactive and radioactive gallium, a test was carried out to assess that the selected method, i.e., the direct labelling of the liposome, was suitable for the chosen nanostructure. Two different approaches to incorporate gallium into liposomes were studied. In the first one, the preformed (Ga)NOTA-OL complex was mixed with POPC, and in the other one, NOTA-OL/POPC liposomes were reacted with the Ga salt.

### 3.4. (Ga)NOTA-OL/POPC Liposomes: Preparation and Characterization

NOTA-OL and (Ga)NOTA-OL were thus separately incorporated into POPC-based liposomes using the thin film protocol. It is worth noting that, differently from NOTA-OL, (Ga)NOTA-OL did not easily dissolve in chloroform due to its higher polarity.

**Figure 3:**
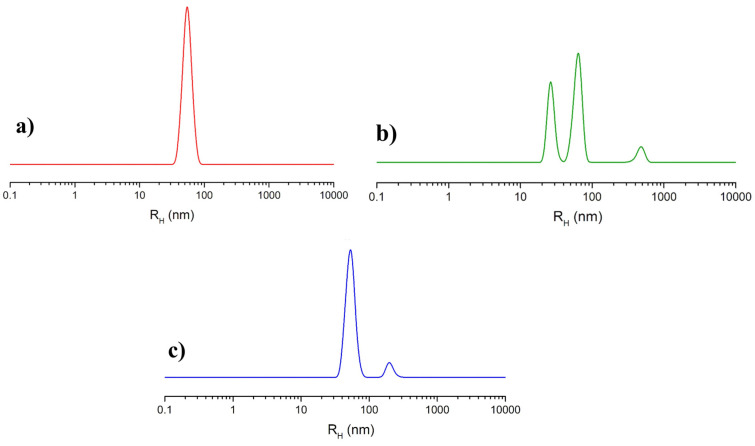
Hydrodynamic radius distribution function obtained through DLS measurements for the prepared liposome formulations: (**a**) extruded NOTA-OL/POPC 5:95 liposomes; (**b**) (Ga)NOTA-OL/POPC 5:95 liposomes obtained with preformed (Ga)NOTA-OL complex; (**c**) extruded NOTA-OL/POPC + Ga liposomes, formed by the addition of Ga(NO_3_)_3_ to preformed 1 mM NOTA-OL/POPC 5:95 liposomes.

The analysis of NOTA-OL/POPC 5:95 liposomes by dynamic light scattering (DLS) measurements showed a single monodisperse population of ca. 50 nm in hydrodynamic radius (Figure 3a), as in the case of pure POPC [35].

**Figure 4:**
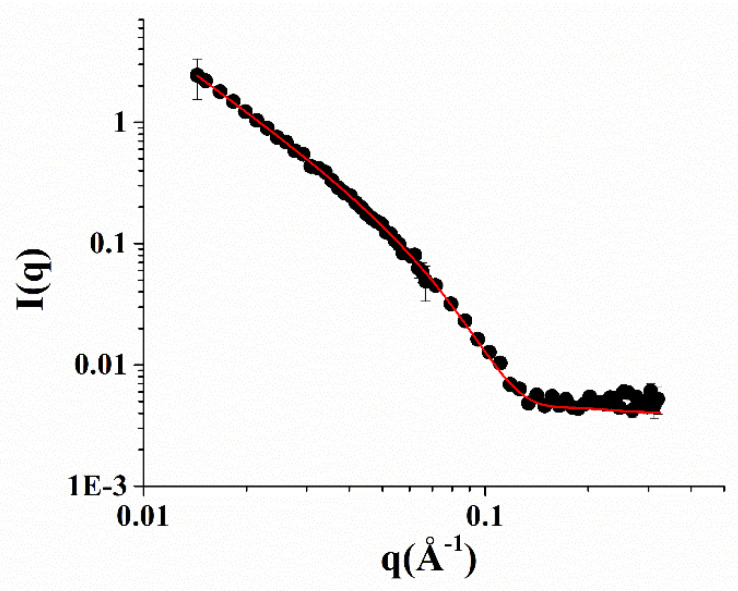
SANS profiles of the NOTA-OL/POPC 5:95 liposomes. Experimental data are represented as black full circles, while the best fitting curve according to a vesicles model is shown as a red line.

Small-angle neutron scattering (SANS) measurements of the NOTA-OL/POPC 5:95 system confirmed the presence of liposomes of about a 50 nm radius formed by a lipid bilayer 4 nm thick (Figure 4).

(Ga)NOTA-OL/POPC 5:95 liposomes, incorporating the preformed (Ga)NOTA-OL complex, were prepared through two different procedures. In one case, the (Ga)NOTA-OL complex dissolved in chloroform was mixed with POPC solution in order to obtain the desired 5:95 NOTA-OL:POPC molar ratio prior to solvent evaporation and thin film formation. In the other case, a thin film of POPC was prepared by evaporation of the organic solvent and the sample was then hydrated with an aqueous solution containing the (Ga)NOTA-OL complex, strictly maintaining the desired 5:95 molar ratio between the NOTA derivative and the POPC lipid and the final 1 mM lipid concentration. However, in both cases, DLS analysis showed the presence of several populations, with the main ones being the expected 50 nm hydrodynamic radius (typical of POPC-based liposomes) and a second one with a hydrodynamic radius of 25 nm, which could be due to elongated/rod-like micelles (as an example, the DLS profile of samples obtained using the second procedure is reported in Figure 3b). The observed DLS profile could be a consequence of the higher polarity of (Ga)NOTA-OL with respect to NOTA-OL, determining its low solubility in chloroform and favoring its self-assembly into small aggregates rather than its incorporation into POPC liposomes.

These results showed that pre-formed (Ga)NOTA-OL complexes could not be successfully used for the preparation of (Ga)NOTA-OL/POPC liposomes. Therefore, we tested an alternative procedure, i.e., the direct labelling method on NOTA-OL/POPC liposomes, which is preferred for its faster applicability considering the short half-life of the radionuclide.

Starting from preformed 1 mM NOTA-OL/POPC 5:95 liposomes, the amount of Ga(NO_3_)_3_ required to obtain the desired 1:1 mol ratio with NOTA-OL was added to the mixture. After acidification to reach pH 3–4, the reaction mixture was left under stirring at a moderately high temperature (50–60 °C) and then at r.t. overnight. The DLS analysis of this sample showed a single population of ca. 50 nm in hydrodynamic radius, with no additional peaks due to smaller aggregates (Figure 3c).

These results showed that the direct labelling of the pre-formed NOTA-OL/POPC liposomes is a suitable procedure to obtain stable and essentially monodisperse gallium-containing nanosystems.

### 3.5. Set-Up of Labelling Reaction

To optimize the reaction conditions, in the previously performed experiments, NOTA-OL was used either in excess or in a 1:1 ratio with gallium. The NOTA-OL/POPC formulations, prepared in water at a 1 mM concentration with a 5% in mol of the target NOTA-OL, contained an overall concentration of the chelating agent lower than that of NOTA-OL alone in the preliminary tests described in Section 3.2 and Section 3.3. The concentration of NOTA-OL in the final mixture with POPC was then further reduced by dilution due to the successive addition of the ^68^Ga solution, and thus in the radiolabelling experiments, it was eventually lower than 50 μM. Therefore, we verified if there was a lower limit of the NOTA-OL concentration below which the labelling reaction proved to be unsuccessful. For this purpose, we performed a series of reactions using different NOTA-OL concentrations in the μM range. The complexation reactions were monitored by HPLC analysis on aliquots of the crude mixture and the corresponding results are reported in Table 1.

The obtained data indicated that the labelling reaction had a reasonable radiochemical yield only if the final concentration of NOTA-OL in the solution was higher than 25 μM. Similar results suggested that NOTA-OL/POPC 5:95 liposomes could not be diluted more than twice. To overcome this limitation, we prepared liposome formulations containing an increased amount of NOTA-OL, i.e., 1 mM NOTA-OL/POPC 10:90 and 2 mM NOTA-OL/POPC 10:90 liposome formulations, and analyzed both formulations by DLS measurements.

**Figure 5:**
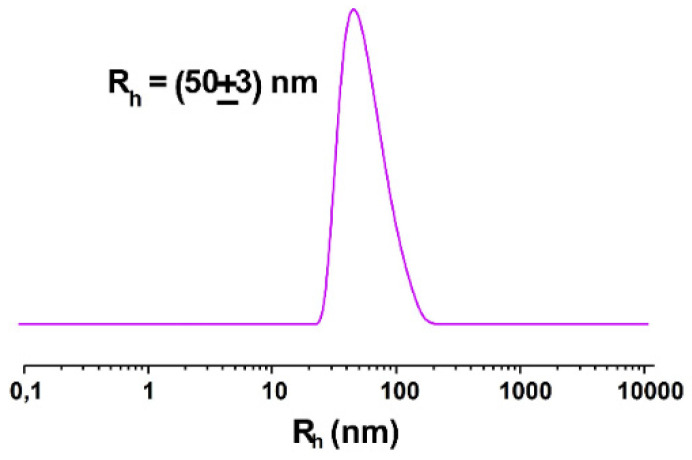
Hydrodynamic radius distribution function obtained through DLS measurements for 1 mM extruded NOTA-OL/POPC 10:90 liposomes.

In the first preparation, in which the overall lipid concentration was kept at 1 mM and the molar content of NOTA-OL was increased from 5 to 10%, the DLS profile showed the typical hydrodynamic distribution of POPC-based liposomes, with a unique population with a size of about 50 nm (Figure 5).

On the contrary, in the second type of NOTA-OL/POPC formulation, in which not only the molar content of NOTA-OL, but also the total lipid concentration increased (the first value, from 5 to 10%; the second one, from 1 to 2 mM, respectively), DLS analysis showed multiple populations, with hydrodynamic radii higher than that of POPC (data not shown). Thus, this kind of system was not further investigated.

On this basis, we selected NOTA-OL/POPC liposomes with a final lipid concentration of 1 mM and containing 10% in mol of the lipophilic NOTA derivative as the best system for the successive radiolabelling procedure.

### 3.6. Synthesis of [^68^Ga]NOTA-OL/POPC

**Figure 6:**
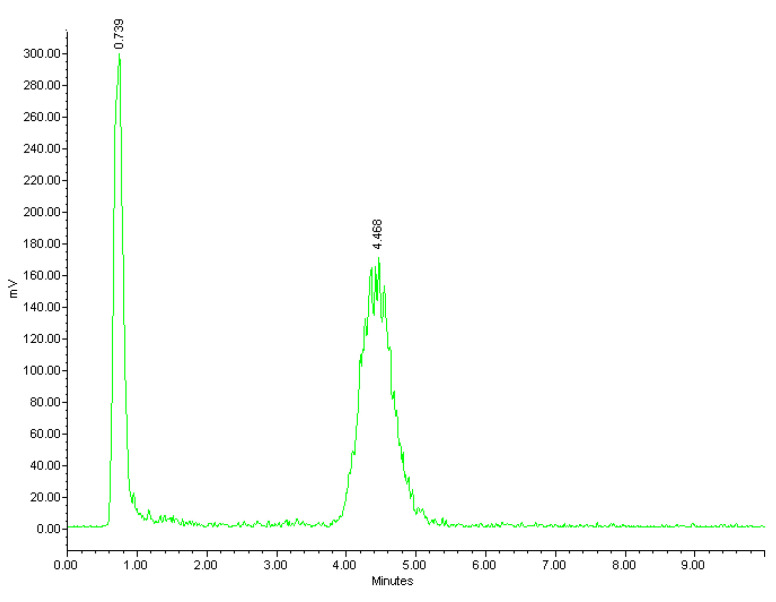
Radio-HPLC chromatogram of the reaction mixture of [^68^Ga]NOTA-OL/POPC liposomes.

The radiolabelling of NOTA-OL/POPC 10:90 liposomes was performed working at a concentration of NOTA-OL in the 50–70 μM range (*v/v* liposome/^68^Ga^3+^ solution ~2.5). The reaction conditions (temperature, stoichiometry, pH, and time) were varied in order to identify the optimal conditions for the ^68^Ga complexation reaction. At pH in the range 4–5, a maximum labelling yield of 50% was obtained; this value decreased to 40% using solutions at pH 5. Low pH values (1–2) led to a low radiochemical yield (~20%) while at pH 3, yields were around 60%. Regarding temperatures, at values below 60 °C, the reaction did not take place, while temperatures >70° did not lead to an improved reaction yield. The best results were thus obtained using temperatures in the range 60–70 °C and maintaining the pH value in the range 3–4. Under these conditions, the radiochemical yield approached 70%. The efficacy of the labelling reactions and the radiochemical yield were assessed by HPLC using a reverse phase column. Although for liposomes other types of stationary phases would be more suitable (a variety of size exclusion and cationic exchange HPLC columns were also tested during this study, data not shown), this experimental set-up allowed identification of both the unreacted ^68^Ga and the labelled liposomes, providing real-time information about the outcome of the radiolabelling reaction. A typical HPLC chromatogram of the crude reaction mixture is shown in Figure 6, where the peaks of free Gallium-68 and [^68^Ga]NOTA-OL/POPC are detectable at 0.7 and 4.5 min., respectively.

A similar result was obtained from radio-TLC analysis, showing two well-separated peaks corresponding to unreacted ^68^Ga^3+^ (eluted with the solvent front) and labelled liposomes (at the baseline).

To quantitatively remove the unreacted free ^68^Ga(III) and recover the [^68^Ga]NOTA-OL/POPC liposomes, a purification method was set up using a cationic exchange cartridge eluted with a solution of saline and acetone. The efficacy of separation was evaluated by analyzing each eluted fraction by both radio-HPLC and radio-TLC.

This test of radiolabelling of NOTA-OL/POPC liposomes demonstrated the feasibility of the selected formulations for an efficient labelling with the ^68^Ga radionuclide. Therefore, this study represents a valuable basis for setting up further studies on animals to evaluate the in vivo biodistribution of the optimized radiolabelled nanostructure by PET.

## 4. Conclusions

In the literature, several POPC-based liposomes have been prepared and characterized for use as effective carriers for the delivery of a variety of therapeutic agents. With the ultimate aim of evaluating the biodistribution, accumulation, and clearance of POPC-based nanosystems for PET in future animal studies, in this work, we co-formulated POPC with a new designed lipophilic NOTA derivative able to coordinate ^68^Ga(III), obtaining stable liposomes of ca. 50 nm size, characterized by DLS and SANS techniques. The NOTA-OL/POPC liposomes were then tested in several labelling procedures with the ^68^Ga radionuclide. The best labelling method was selected, and the reaction conditions optimized to obtain a significant (>70%, estimated by HPLC) radiochemical yield. These results demonstrated the feasibility of our approach for labelling POPC liposomes with ^68^Ga, stably complexed to a lipophilic NOTA analogue, derivatized with oleic acid, and easily synthesized in five reaction steps.

## Figures and Tables

**Table 1:** Radiochemical yield (determined by radio-HPLC analysis of the crude product) of NOTA-OL labelling reaction with Gallium-68 at different concentrations of the substrate.

Concentration of NOTA-OL in the Bulk	Radiochemical Yield (ndc) *
0.1 μM	0
1 μM	0
5 μM	0
10 μM	0
20 μM	<10%
25 μM	60–70%
85 μM	>80%

* ndc: not decay corrected.

## Data Availability

Not applicable.

## Supplementary Materials

The following are available online, Table S1. Selected radionuclides used in clinical PET analysis, their half-lives, decay properties, and production mode. Figure S1. HPLC profile of pure NOTA-OL. Figure S2. MALDI-TOF spectrum (positive ions) of NOTA-OL. Figure S3. UV-vis absorption spectra of the commercially available NODA-Ga(tBu)_3_ and of NOTA-OL. Figure S4. Surface tension measurements for the binary mixture aqueous-NOTA-OL as a function of the molar concentration of NOTA-OL. Figure S5. MALDI-TOF spectrum (positive ions) of the crude reaction mixture of NOTA-OL with Ga(NO_3_)_3_. Supplemental data to Materials and Methods: NMR and MALDI characterization of NOTA-OL and its synthetic precursors.

## Sample Availability

No samples are available from the authors.

## Abbreviations

AcOEt	ethyl acetate
COSY	correlation spectroscopy
DCM	dichloromethane
DHB	2,5-dihydroxybenzoic acid
DIPEA	*N*,*N*-diisopropylethylamine
DLS	dynamic light scattering
DMAP	4-(*N*,*N*-dimethylamino)pyridine
DOTA	(1,4,7,10-tetraazacyclododecane-1,4,7,10-tetraacetic acid)
HATU	1-[bis(dimethylamino)methylene]-1*H*-1,2,3-triazolo [4,5-*b*]pyridinium 3-oxide hexafluoro-phosphate
HSQC	heteronuclear single quantum correlation
MALDI	matrix-assisted laser desorption ionization
MeOH	methanol
MMT	4-monomethoxytrityl
MMT-Cl	4-methoxytriphenylmethyl chloride
MS	mass spectrometry
NMR	nuclear magnetic resonance
NOTA	1,4,7-triazacyclononane-1,4,7-triacetic acid
PET	positron emission tomography
ppm	parts per million
*R_f_*	retention factor
RP-HPLC	reversed phase high performance liquid chromatography
*t*Bu	*tert*-butyl
TCA	trichloroacetic acid
TEA	triethylamine
TLC	thin layer chromatography
TOF	time of flight
*t*_R_	retention time
UV	ultraviolet spectroscopy
