# Supplementary material for: Synthesis and Characterization of Multifunctional Nanovesicles Composed of POPC Lipid Molecules for Nuclear Imaging
Source: Molecules. 2021 Oct 30;26(21):6591. doi: 10.3390/molecules26216591 (PMC8587727; doi:10.3390/molecules26216591)
Supplement: Supplementary file 1 [file molecules-26-06591-s001.zip › molecules-1430292-supplementary.pdf]

molecules

Supplementary Material: Synthesis and Characterization of Multifunctional Nanovesicles Composed of POPC Lipid Molecules for Nuclear Imaging

Debora Petroni [1*], Claudia Riccardi [2], Domenico Cavasso [2,3], Irene Russo Krauss [2,3], Luigi Paduano [2,3]Daniela Montesarchio [2] and Luca Menichetti [1]

[1] Institute of Clinical Physiology-National Research Council, Via Moruzzi 1, I-56124 Pisa, Italy; debora.petroni@ifc.cnr.it; luca.menichetti@ifc.cnr.it
[2] Department of Chemical Sciences, University of Naples Federico II, Via Cintia 21, I-80126 Naples, Italy; Department of Chemical Sciences, University of Naples Federico II, Via Cintia 21, I-80126 Naples; claudia.riccardi@unina.it; domenico.cavasso@unina.it, irene.russokrauss@unina.it; luigi.paduano@unina.it daniela.montesarchio@unina.it
[3] CSGI - Consorzio per lo Sviluppo dei Sistemi a Grande Interfase, Florence, Italy

Table of contents

Table S1. Selected radionuclides used in clinical PET analysis, their half-lives, decay properties and production mode. pag. 2

Figure S1. HPLC profile of pure NOTA-OL. pag. 3

Figure S2. MALDI-TOF spectrum (positive ions) of NOTA-OL. pag. 3

Figure S3. UV-vis absorption spectra of NODA-Ga(tBu)$_3$ and of NOTA-OL. pag. 4

Figure S4. Determination of the cmc of NOTA-OL in aq. solution. pag. 5

Figure S5. MALDI-TOF spectrum of the crude reaction mixture of NOTA-OL with Ga(NO$_3$)$_3$. pag. 6

Supplemental data to Materials and Methods: NMR and MALDI characterization of NOTA-OL and its synthetic precursors pag. 7

Table S1. Selected radionuclides used in clinical PET analysis, their half-lives, decay properties and production mode.

Radionuclide	$T_{1/2}$	Decay (%)	Production
^{18}F	109.7 min	β^+(96.7)	^{18}O (p,n) ^{18}F
		EC (0.1)	
^{68}Ga	67.7 min	β^+(89)	$^{68}Ge/^{68}Ga$ generator
		EC (11)	
^{64}Cu	12.7 h	β^+(17)	^{64}Ni (p,n) ^{64}Cu
		EC (44)	
^{124}I	4.18 d	β^+(23)	^{124}Te (p,n) ^{124}I
		EC (77)	^{124}Te (d,n) ^{124}I
^{86}Y	14.7 h	β^+(33)	^{86}Sr (p,n) ^{86}Y
		EC (66)	
^{89}Zr	3.3 d	β^+(23)	^{89}Y (p,n) ^{89}Zr
		EC (77)	
^{76}Br	3.3 d	β^+(55)	^{76}Se (p,n) ^{76}Br
		EC (45)	^{76}Se (d,2n) ^{76}Br

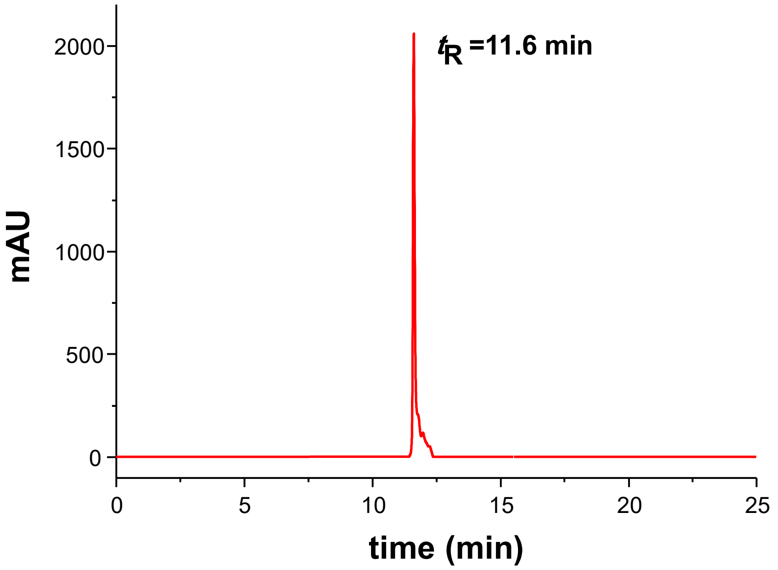

Figure S1. HPLC profile of pure NOTA-OL.

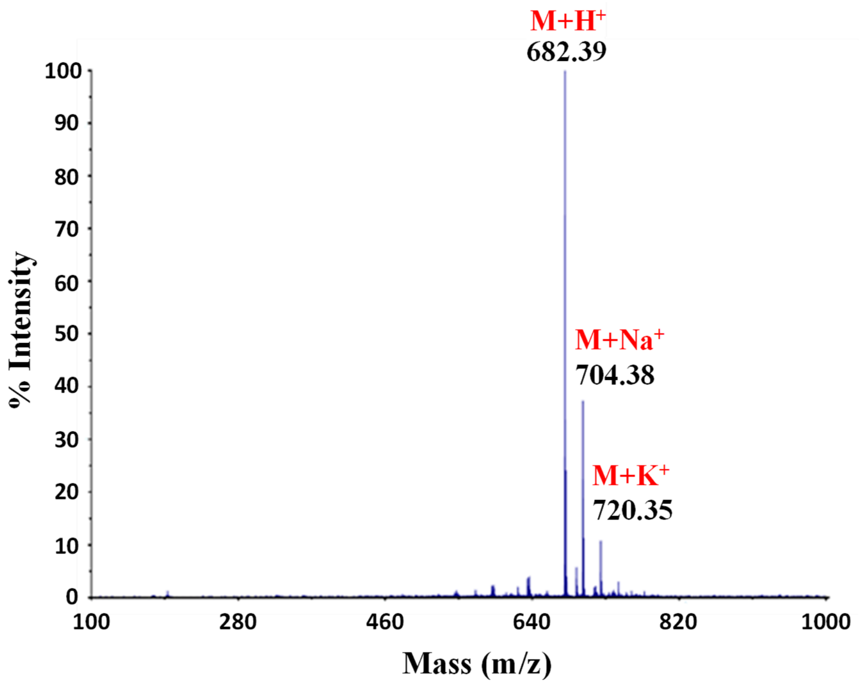

Figure S2. MALDI-TOF spectrum (positive ions) of NOTA-OL.

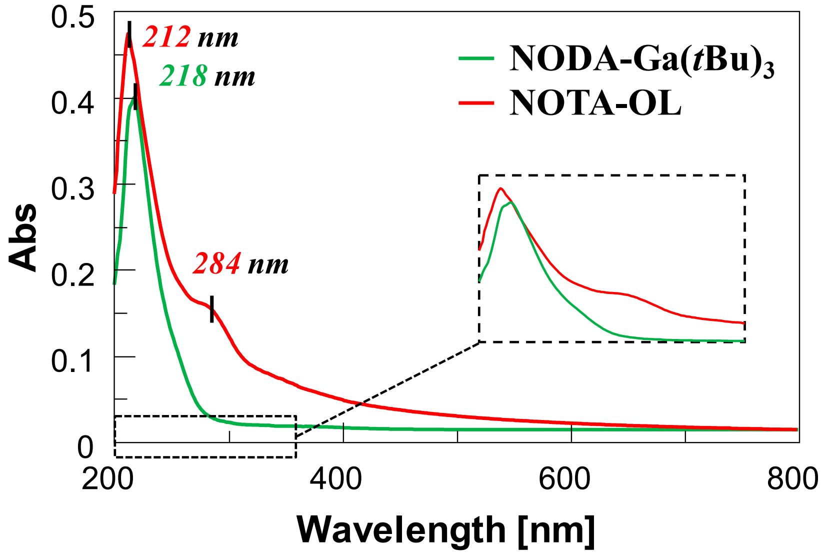

Figure S3. Overlapped UV-vis absorption spectra of the commercially available NODA-Ga(tBu)3 and of the target NOTA-OL (green and red lines, respectively) at 100 μM concentration in CH_3CN. The inset represents an enlargement of the 200-350 nm area.

Determination of the cmc of NOTA-OL in aq. solution

The self-aggregation behaviour of NOTA-OL was investigated in aqueous solution. Thanks to its amphiphilic character, it can form supramolecular assemblies in aqueous mixtures, driven by hydrophobic interactions among the oleyl chains. With this information, in the liposome preparation, we could discard concentration conditions under which the self-micellization of the lipophilic macrocycle was the prevailing process, even in the presence of high concentrations of POPC.

The cmc of NOTA-OL in aqueous was determined by surface tension measurements. The surface tension data relative to the binary system aqueous mixture-NOTA-OL, reported as a function of the amphiphile concentration (m) are shown in **Figure S4**.

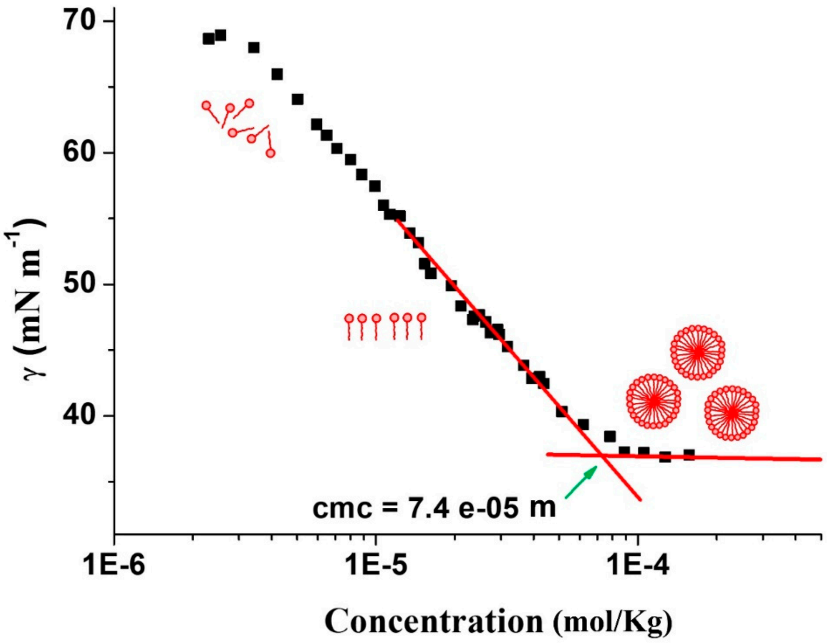

Figure S4. Surface tension measurements for the binary mixture aqueous-NOTA-OL as a function of the molar concentration of NOTA-OL.

With the increase of NOTA-OL concentration, the surface tension (γ) decreased, and consequently, the macrocyclic derivative tended to segregate at the air-solution interface. Upon further increasing the concentration, γ reached a constant value, *i.e.* 34.5 (± 0.5) mN m^{-1}. This evidence indicated that NOTA-OL concentration at the interface did not increase and all the added molecules only contributed to the formation of micelles in the bulk solution. Thus, the molarity at which a constant γ value was reached provided the NOTA-OL cmc, estimated to be equal to $\cong$ 7.4 ·x 10^{-5} m. This result indicated that NOTA-OL could be considered as a unimer in solutions with NOTA-OL concentrations up to μm concentration, and as an aggregate in solutions with concentrations of at least one order of magnitude higher.

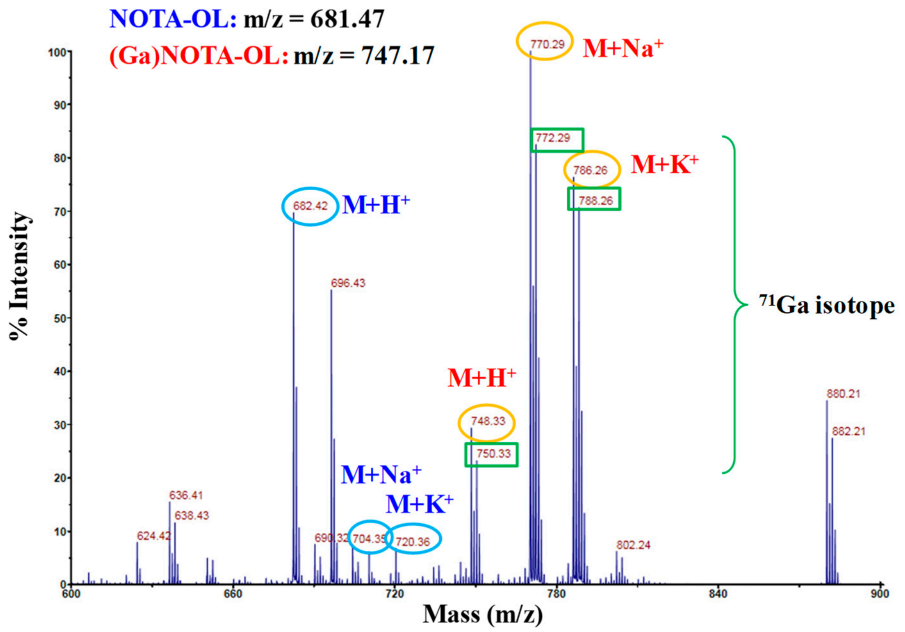

Figure S5. MALDI-TOF spectrum (positive ions) of the crude reaction mixture of NOTA-OL with $Ga(NO_3)_3$.

Materials and Methods

In order to easily identify the proton and carbon signal values of the compounds in NMR spectra, letters and numbers were assigned to the structures of MMT and NOTA moieties, as shown in the following pictures.

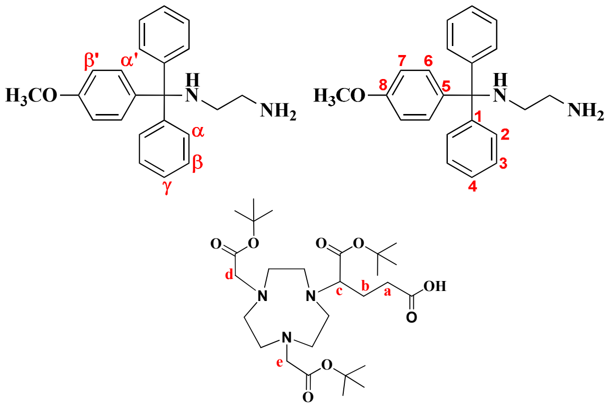

The oleic acid chain was indicated with progressive numbers (1′-18′) starting from the carbonyl group.

The following abbreviations were used to explain the multiplicities: s = singlet; d = doublet; t = triplet; q = quartet; m = multiplet; b = broad.

N-1-[(4-methoxyphenyl)diphenylmethyl]ethane-1,2-diamine (2)

R_f = 0.6 (DCM/MeOH, 9:1, v/v).

^{1}H NMR (400 MHz, $CDCl_3$): *δ* 7.48 (d, *J* = 7.7 Hz, 4H, 4x *H*α); 7.39 (d, *J* = 8.6 Hz, 2H, 2x *H*α′); 7.26 (t, *J* = 7.4 and 7.6 Hz, 4H, 4x *H*β); 7.17 (d, *J* = 7.1 Hz, 2H, 2x *H*γ); 6.80 (d, *J* = 8.6 Hz, 2H, 2x Hβ′); 3.74 (s, 3H, -OCH_3); 2.78 (t, *J* =5.8 Hz, 2H, -CH_2NH_2); 2.22 (t, *J* =5.8 Hz, 2H, -CH_2NH MMT).

^{13}C NMR (100 MHz, $CDCl_3$): *δ* 157.6 (*C*-8); 146.1 (2x *C*-1); 138.0 (*C*-5); 129.5 (2x *C*-6); 128.3 (4x *C*-2); 127.5 (4x *C*-3); 125.9 (2x *C*-4); 112.8 (2x *C*-7); 69.9 (quaternary carbon of MMT); 54.8 (-OCH_3); 46.0 (-CH_2NH_2); 42.4 (-CH_2NH MMT).

N-2-[(4-methoxyphenyl)diphenylmethylamino]ethyl oleamide (3)

R_f = 0.6 (*n*-hexane/AcOEt 6:4, v/v).

^{1}H NMR (400 MHz, $CDCl_3$): *δ* 7.44 (d, *J* = 7.6 Hz, 4H, 4x *H*α); 7.35 (d, *J* = 8.6 Hz, 2H, 2x *H*α′); 7.27 (t, *J* = 7.4 and 7.5 Hz, 4H, 4x *H*β); 7.19 (d, *J* = 7.1 Hz, 2H, 2x *H*γ); 6.81 (d, *J* = 8.5 Hz, 2H, 2x Hβ′); 5.98 (bs, 1H, N*H*); 5.35 (s, 2H, C*H*-9′, 10′); 3.77 (s, 3H, -OCH_3); 3.35 (bd, 2H, -CH_2NH oleic); 2.33 (bt, 2H, -CH_2NH MMT); 2.18 (t, *J* = 7.5 and 7.6 Hz, 2H, CH_2-2′); 2.01 (overlapped signals, 4H, 2x CH_2-8′, 11′); 1.66-1.63 (m, 2H, CH_2-3′); 1.31-1.28 (overlapped signals, 20H, 10x CH_2-4′, 5′, 6′, 7′, 12′, 13′, 14′, 15′, 16′, 17′); 0.89 (t, *J* = 6.0 and 6.8 Hz, 3H, CH_3-18′).

^{13}C NMR (100 MHz, $CDCl_3$): *δ* 173.3 (*C*=O oleic); 158.0 (*C*-8); 145.6 (2x *C*-1); 137.5 (*C*-5); 129.9 (overlapped signals, 2x CH-9′, 10′); 129.7 (2x *C*-6); 128.3 (4x *C*-2); 127.8 (4x *C*-3); 126.4 (2x *C*-4); 113.1 (2x *C*-7); 70.5 (quaternary carbon of MMT); 55.1 (-OCH_3); 43.7 (-CH_2NH oleic); 39.5 (-CH_2NH MMT); 36.8 (CH_2-2′); 31.8, 29.6, 29.4, 29.2, 29.0, 22.5 (overlapped signals, 10x CH_2-, 4′, 5′, 6′, 7′, 12′, 13′, 14′, 15′, 16′, 17′); 27.1 (overlapped signals, 2x CH_2-8′, 11′); 25.7 (CH_2-3′); 14.0 (CH_3-18′).

MALDI-MS (positive ions): calculated for $C_{40}H_{56}N_2O_2$, 596.43; found m/z: 347.23 [M-MMT+Na^+]; 363.20 [M-MMT+K^+].

N-(2-aminoethyl)oleamide (4)

R_f = 0.4 (DCM/MeOH, 9:1, v/v).

^{1}H NMR (400 MHz, $CDCl_3$): *δ* 6.55 (bs, 1H, N*H*C=O oleic); 5.32 (s, 2H, C*H*-9′, 10′); 3.27 (bs, 2H, -CH_2NH oleic); 3.06 (bs, 2H, NH_2); 2.90 (bt, 2H, -CH_2NH_2); 2.19 (t, *J* = 7.4 and 7.3 Hz, 2H, CH_2-2′); 1.98 (overlapped signals, 4H, 2x CH_2-8′, 11′); 1.60 (m, 2H, CH_2-3′); 1.28-1.19 (overlapped signals, 20H, 10x CH_2-4′, 5′, 6′, 7′, 12′, 13′, 14′, 15′, 16′, 17′); 0.86 (bt, 3H, CH_3-18′).

^{13}C NMR (100 MHz, $CDCl_3$): *δ* 174.7 (*C*=O oleic); 129.5, 129.4 (overlapped signals, 2x *C*H-9′, 10′); 39.9 (-*C*H$_2$NH oleic); 36.7 (-*C*H$_2$NH$_2$); 36.1 (*C*H$_2$-2′); 31.5, 29.4, 28.9, 22.3 (overlapped signals, 10x *C*H$_2$-4′, 5′, 6′, 7′, 12′, 13′, 14′, 15′, 16′, 17′); 26.9 (overlapped signals, 2x *C*H$_2$-8′, 11′); 25.3 (*C*H$_2$-3′); 13.8 (*C*H$_3$-18′).

MALDI-MS (positive ions): calculated for $C_{20}H_{40}N_2O$, 324.31; found m/z: 347.17 [M+Na$^+$]; 363.19 [M+K$^+$].

(Z)-di-*tert*-butyl 2,2'-[7-(1-*tert*-butoxy-5-2-oleamidoethylamino-1,5-dioxopentan-2-yl)-1,4,7-triazonane-1,4-diyl]di-acetate (5)

^{1}H NMR (400 MHz, $CDCl_3$): *δ* 6.48 (s, 1H, N*H*); 5.33 (s, 2H, C*H*-9′, 10′); 3.75 (m, 2H, -CH_2NH oleic); 3.65 (overlapped signals, 4H, CH_2-d and CH_2-e); 3.38 (overlapped signals, 3H, C*H*-c, -CH_2NH oleic); 3.23-2.80 (overlapped signals, 14H, -NCH_2CH_2N- of the macrocycle, CH_2NH); 2.38 (m, 2H, CH_2-a); 2.20 (bt, 2H, CH_2-2′); 1.99 (overlapped signals, 6H, 2x CH_2-8′, 11′, CH_2-b); 1.59 (m, 2H, CH_2-3′); 1.47 (s, 27H, CH_3 of -*t*Bu); 1.28-1.26 (overlapped signals, 20H, 10x CH_2-4′, 5′, 6′, 7′, 12′, 13′, 14′, 15′, 16′, 17′); 0.87 (bt, 3H, CH_3-18′).

^{13}C NMR (100 MHz, $CDCl_3$): *δ* 174.6 (overlapped signals, *C*=O NOTA, *C*=O oleamide); 172.8 (overlapped signals, 3x *C*OO*t*Bu); 129.8, 129.7 (2x *C*H-9′, 10′); 82.8 (overlapped signals, 3x quaternary carbons of *t*Bu); 63.8 (*C*H-c); 55.9 (overlapped signals, *C*H$_2$-d and *C*H$_2$-e); 54.3, 53.9, 51.7 (overlapped signals, -NCH$_2$CH$_2$N- of the macrocycle); 39.9 (-*C*H$_2$NH oleamide); 39.5 (-CH$_2$NH NOTA); 36.4 (*C*H$_2$-2′); 32.7 (*C*H$_2$-a); 31.8, 29.7, 29.5, 29.2, 29.1, 22.6 (overlapped signals, 10x *C*H$_2$-4′, 5′, 6′, 7′, 12′, 13′, 14′, 15′, 16′, 17′); 27.9 (*C*H$_3$ of -*t*Bu); 27.1 (overlapped signals, 2x *C*H$_2$-8′, 11′); 25.6 (*C*H$_2$-b); 25.3 (*C*H$_2$-3′); 14.0 (*C*H$_3$-18′).

MALDI-MS (positive ions): calculated for $C_{47}H_{87}N_5O_8$, 849.66; found *m*/*z*: 850.60 [M+H$^+$]; 872.58 [M+Na$^+$]; 888.55 [M+K$^+$].

Synthesis of NOTA-OL

^{1}H NMR (400 MHz, $CDCl_3$): *δ* 5.33 (s, 2H, C*H*-9′, 10′); 4.01-3.36 (overlapped signals, 5H, CH_2-d, CH_2-e, C*H*-c,); 2.97 (overlapped signals, 4H, -CH_2NH oleic, -CH_2NH NOTA); 2.85 (overlapped signals, 14H, -NCH_2CH_2N- of macrocycle, CH_2-2′); 2.40 (m, 2H, CH_2-a); 2.01-1.83 (overlapped signals, 6H, 2x CH_2-8′, 11′, CH_2-b); 1.56 (m, 2H, CH_2-3′); 1.25-1.23 (overlapped signals, 20H, 10 x CH_2-4′, 5′, 6′, 7′, 12′, 13′, 14′, 15′, 16′, 17′); 0.87 (bt, 3H, CH_3-18′).

^{13}C NMR (100 MHz, $CDCl_3$): *δ* 175.3 (3x COOH); 172.0 (*C*=O NOTA); 171.4 (*C*=O oleic); 129.9, 128.2 (overlapped signals, 2x *C*H-9′, 10′); 65.6 (*C*H-c); 56.3 (overlapped signals, *C*H$_2$-d and *C*H$_2$-e); 53.8, 52.2, 49.4 (overlapped signals, -N*C*H$_2$*C*H$_2$N- of macrocycle); 39.2 (-*C*H$_2$NH oleic); 38.5 (*C*H$_2$-2′); 36.5 (-*C*H$_2$NH NOTA); 34.6 (*C*H$_2$-a); 31.8, 29.7, 29.6, 29.4, 29.2, 22.6 (overlapped signals, 10x *C*H$_2$-4′, 5′, 6′, 7′, 12′, 13′, 14′, 15′, 16′, 17′); 27.1 (overlapped signals, 2x *C*H$_2$-8′, 11′); 26.6 (*C*H$_2$-3′); 24.5 (*C*H$_2$-b); 14.0 (*C*H$_3$-18′).

MALDI-MS (positive ions): calculated for $C_{35}H_{63}N_5O_8$, 681.47; found *m*/*z*: 682.39 [M+H$^+$]; 704.38 [M+Na$^+$]; 720.35[M+K$^+$].

Preparation of the non-radioactive (Ga)NOTA-OL complex

MALDI-MS (positive ions): calculated for $C_{35}H_{60}N_5O_8Ga$, 747.17; found m/z: 748.33 [M+H$^+$]; 770.29 [M+Na$^+$]; 786.26 [M+K$^+$].
